# Woody Species Composition, Structure, and Status of Regeneration in Pugnido Forest, Gambella Region, Western Ethiopia

**DOI:** 10.1155/2024/3961434

**Published:** 2024-04-04

**Authors:** Getinet Masresha, Yirgalem Melkamu, Getachew Mulu

**Affiliations:** University of Gondar, Department of Biology, P.O. Box. 196, Gondar, Ethiopia

## Abstract

Ethiopia is a biodiversity hotspot area with a high concentration of plant species that play countless roles in ecosystem resilience and mitigating the effects of climate change. However, forest resources are degrading at an alarming rate due to different anthropogenic factors. Pugnido Forest, which is among Ethiopia's greatest woodland sections, also faced a similar challenge. Thus, this study was intended to assess the composition, structure, and regeneration status of woody plant species in Pugnido Forest, West Ethiopia. Eighty main plots sized 20 × 20 m were sampled systematically on six east-west-oriented line transects, which are parallel to each other and 500 m apart. At the four corners and the center of the main plots, five subplots sized 2 m × 2 m were laid to collect seedling and sapling information. Individual woody species were identified and then recorded per plot. The forest structure and regeneration status data were recorded and analyzed using structural parameters. Communities were identified via cluster analysis. A comparison of diversity and composition among communities was made using the Shannon–Wiener diversity and Sorensen's similarity coefficients, respectively. A total of 106 woody species included in 40 families were documented. Fabaceae stands first in the species-rich family (23.6%), and Moraceae stands second (8.5%). Overall Shannon–Wiener diversity and evenness of species were 4.41 and 0.93, respectively. Four communities were generated from cluster analysis. Sorensen's similarity value among communities ranged from 42% to 59%. The forest's density and basal area were 5298.8 stems·ha^−1^ and 82.5 m^2^·ha^−1^, respectively. The trend in frequency and density classes showed a decreasing number of species towards higher classes. Diameter at breast height (DBH), height, and basal area analyses revealed a normal J-shaped pattern. Several species had a smaller importance value index (IVI) value. Density ratios of juveniles to adult plants indicated good regeneration status. Species with small IVI values and few or no seedlings require conservation priority. Top priority must be given to conserving species with low IVI values and few or no seedlings.

## 1. Introduction

Ethiopia is among the world's nations with the greatest diversity of flora and fauna [1]. The variation in topography, climate, and edaphic factors enables the country to be the owner of diverse vegetation from alpine to desert ecosystems [2]. It is Africa's fifth largest floral diversity country with nearly 6027 species of higher plants, of which about 780–840 (10%) are endemic [3]. However, currently, this diverse vegetation, predominantly forest resources, is severely threatened due to anthropogenic effects associated with rapid population growth: the need for more farmlands, grazing lands, settlement (urbanization), increasing fuelwood demand, climatic changes, and other related problems [4]. At this moment, only a few forest patches are remaining and most of these are in a secondary state of development or represent various stages in the development of forests [5]. This eventually results in ecosystem instability and reduced availability of various forest goods and services.

Pugnido Forest is one of the remaining forest patches in Ethiopia with rich natural plant resources that provide goods and services essential for the survival and well-being of the surrounding community. Now, the forest is under serious threat due to different manmade pressures. On the contrary, any botanical studies are not conducted in the forest. As a result, floristic assessments are essential for providing information on biodiversity and forest ecosystem functioning [6]. Consequently, the purpose of this investigation was to provide quantitative data on the floristic formation of woody plants and to determine the current population structure and regeneration status of the forest to create attention to feature conservation priorities.

## 2. Materials and Methods

### 2.1. Study Area Description

**Figure 1:**
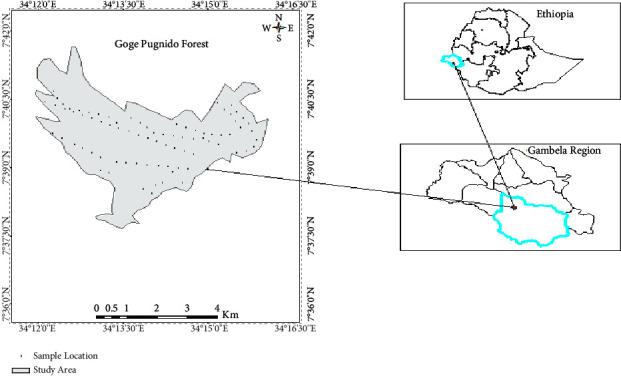
Map of the study area.

**Figure 2:**
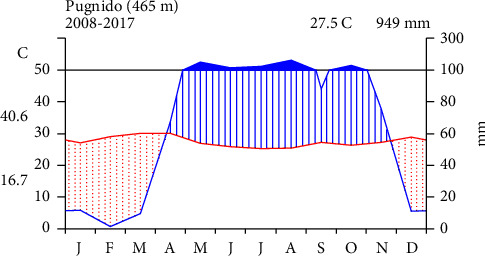
Pugnido meteorological station climate diagram.

The investigation was carried out on Pugnido Forest, one of the largest forest patches, found in Gog District, Anuak Zone of Gambella Regional State, Ethiopia. It is located approximately 846 km west of Ethiopia's capital, Addis Ababa, and covers an area of 2106.48 ha. Geographically, it is located about 7°36′00″ N to 7°42′00″ N latitude and 34°12′00″ E to 34°16′30″ E longitude (Figure 1). The forest is predominantly flat with an elevation difference of 400 to 552 meters above sea level. It is dissected by the Gilo River in the east-west direction and by one main lake known as Lake Tata around the forest's middle part. The area has a three-modal rainfall pattern with annual average rainfall and temperature of 79.2 mm and 27.47°C, respectively (Figure 2).

### 2.2. Data Collection

All vegetation data were collected from the 80 20 × 20 m sized main plots with a 300 m interval in between using a systematic random sampling technique along six transect lines. The transect lines were spaced at 500 m interval and oriented in the east-west direction, which is the longest forest line with relatively maximum altitudinal variation following Kent and Coker [7]. In each plot, every woody species with its individuals was recognized and documented by using their scientific and colloquial names. The plant identification procedure was conducted in situ using taxonomists. Some species that were unable for in situ identification difficulty were identified with the help of the flora of Ethiopia and Eretria using voucher specimens. Five 2 m × 2 m subplots were laid at the four corners and one at the center of the main plots to record the number of saplings and seedlings [8]. Terrain variables (geographical position, altitude, aspect, and slope) were measured at the center of all sample plots using Garmin GPS 65. In each sample plot, the circumferences of all adult woody species were measured at breast height (about 1.3 m) and then later changed to DBH. Woody plants were classified as adult plants if their height was more than 3 meters and their DBH was more than 2.5 cm. Saplings were those with a DBH of less than 2.5 cm and a height of more than 1.5 meters, while seedlings were those with a DBH of less than 2.5 cm and a height of less than 1.5 meters as used by [9]. Species that were found 10 meters away from the plots within the forest were noted just as present; however, they were not taken into account for the data analysis that followed [10].

### 2.3. Data Analysis

#### 2.3.1. Community Classification

Using R statistics software, agglomerative hierarchical cluster analysis was accepted to classify the vegetation into plant community categories based on the abundance of species in 80 sample plots [11]. The similarity ratio and Ward's method were used to verify the likeness function and to diminish the total within the group mean square, respectively [7]. The community categories were also presented in a synoptic table. Greater synoptic values were obtained to identify dominating species that are used to name the communities.

#### 2.3.2. Diversity and Resemblance of Species among Communities

Species variation of communities was determined using the Shannon–Wiener diversity index (H′) as follows:

(1) $$\begin{array}{c} H^{\prime }=-\overset{s}{\underset{i=1}{\sum }}P_{i}in\;P_{i}, \end{array}$$

where H′ = Shannon–Wiener diversity index, *s* = species number, Pi = individual proportion, and ln = natural logarithm to base *n*.

Uniformity was also computed as follows:

(2) $$\begin{array}{c} J=\frac{H^{\prime }}{H^{\prime }\;\max}=\frac{\sum_{i=1}^{s}P_{i}in\;P_{i}}{ins}, \end{array}$$

where *J* = evenness of species, H'max = maximum value of diversity, and *S* = community's species number.

#### 2.3.3. Vegetation Structure

Structural parameters, such as frequency (F), density (D), diameter at breast height (DBH), basal area (BA), and importance value index (IVI), were employed to examine the woody species' vegetative structure.

The formula for F was the number of plots with a species/total quadrates × 100. Then, it was sorted ascendingly and grouped into five frequency classes (in %): 1 (≤20, 2 (21–40), 3 (41–60), 4 (61–80), and 5 (81–100). The distributional pattern of species was ascertained by calculating the percent distribution of individuals within each class. Relative frequency of a species (RF) = plot number with a species/total availability for all species of the sample × 100.

Density (D) was evaluated as individual species number per area sampled in hectare. The density was then divided into seven density classes expressed in percentage: class 1 (≤20), class 2 (20.1–40), class 3 (40.01–60), class 4 (60.01–80), class 5 (80.01–100), class 6 (100.01–120), and class 7 (>120.1–140). The relative density of a species (RD) was calculated as (stands sum of a species/sum of all stands of the sample) × 100.

Every adult woody plant's circumference was measured to determine its diameter at breast height (DBH). It was calculated as circumferences divided by Pi (*π*). Once the trees and shrubs were divided into eight DBH groups: I (≤20 cm), II (20.1–40 cm), III (40.1–60 cm), IV (60.1–80 cm), V (80.1–100 cm), VI (100.1–120 cm), VII (120.1–140 cm), and VIII (≥140.1 cm), their distribution was examined. The structural pattern of the forest patches was assessed by calculating the proportion of individuals in each DBH category.

The height of individual woody species was measured, which was then divided into seven height divisions in meters by assorting in ascending order: class I (≤5), class II (5.01–10), class III (10.1–15), class IV (15.1–20), class V (20.1–25), class VI (25.1–30), and class VII (>30). According to the International Union for Forest Research Organization's (IUFRO) classification scheme, the stratification of woody species was outlined [12]. Three vertical structures were established based on peak height: upper story (>2/3 of the top height), middle story (1/3 to 2/3 of the top height), and lower story (<1/3 of the top height).

Basal area (BA) was calculated as BA=*πd*^2^/4, where *d* = diameter at breast height in meters and *π* = 3.14, which is constant. The BA of woody plants was divided into five BA classes: I (0.0–0.51), II (0.51–2), III (2.01–0.5), IV (2.51−2), and V (>2).

Relative species basal area (RBA) = (species BA/total BA of the sample) × 100.

The importance value index (IVI) was calculated by adding relative density (RD), relative frequency (RF), and relative dominance (RDO). Considering the values of IVI, species were arranged into five IVI classes for management precedence: class 1 (>15), class 2 (10.1–15), class 3 (5.1–10), class 4 (1–5), and class 5 (<1) following Shibru and Balcha [8].

Density ratios between seedlings, saplings, and adults were used to evaluate the regeneration status of woody plants, and then, they were stated as “good, fair, or poor” [13].

## 3. Results

### 3.1. Floristic Composition

**Figure 3:**
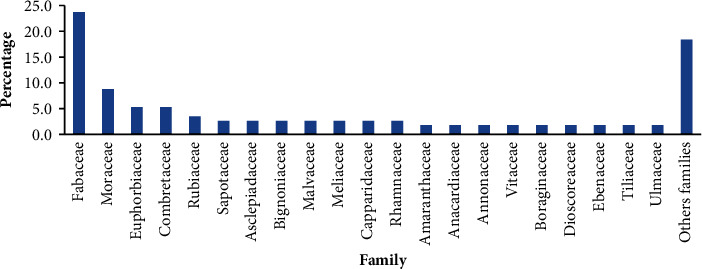
Family dominance of plant species in Pugnido Forest.

Pugnido Forest harbors 106 woody plant species classified into 73 genera and 40 families (Table 1). In growth form, trees make up 72.64% followed by shrubs (18.87%), and the rest (8.49%) were climbers. Family dominancy was taken by Fabaceae (25 species, 23.6%) and then Moraceae (9 species, 8.5%). Combretaceae and Euphorbiaceae were denoted by 6 (5.7%) and 5 (4.7%) species, respectively. Asclepiadaceae, Sapotaceae, and Rubiaceae each were denoted by 4 (3.77%) species. The other 33 families were designated by three, two, or one species (Figure 3; Table 1).

### 3.2. Plant Community Types

**Figure 4:**
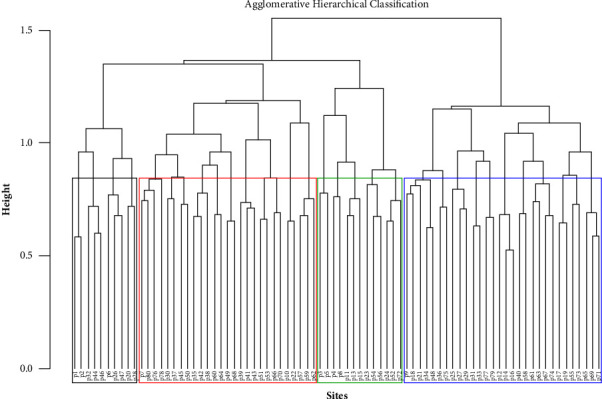
Communities produced by hierarchical agglomeration classification.

In the forest, four plant community types (clusters) were generated using agglomerative hierarchical cluster analysis and named after two species that had a higher synoptic value (Figure 4).

#### 3.2.1. Community I (*Capparis sepiaria*-*Balanites aegyptiaca* Community Type)

It was located in altitudinal ranges of 435 and 454 meters above sea level and embraced the fewest number of plots and species (10 plots and 89 species) than the rest of the communities (Table 2). *Capparis sepiaria* and *Balanites aegyptiaca* were the leading species in the community. *Acacia melanoxylon, Albizia gummifera, Argomuellera macrophylla,* and *Acacia albida* were the leading tree species*. Capparis sepiaria , Bridelia scleroneura, Ziziphus abyssinica, Tapinanthus heteromorphus,* and *Sarcocephalus latifolius* were among the dominant shrub species. Several climber species, *Cissus ruspolii, Phytolacca dodecandra, Dioscorea bulbifera,* and *Dioscorea praehensilis,* were included in this community (Table 3).

#### 3.2.2. Community II (*Albizia lebbeck*-*Dioscorea bulbifera* Community)

It was scattered in between 438 and 457 meters above sea level and contained 13 plots (0.52 ha) and 101 associated species (Table 2). *Albizia lebbeck* and *Dioscorea bulbifera* (climber) were indicator species. Other associated tree species were *Acacia brevispica, Albizia gummifera, Combretum adenogonium, Acacia melanoxylon, Piliostigma thonningii, Capparis tomentosa, Sarcocephalus latifolius,* and *Ziziphus mucronata,* which were dominant shrubs. *Dorstenia barnimiana, Hippocratea africana,* and *Phytolacca dodecandra* were the dominant climbers (Table 3).

*Community III (Adansonia digitata-Bridelia scleroneura community).* This is established between 436 and 462 meters above sea level and is represented by 27 plots (1.08 ha) and 104 associated species (Table 2). The community's primary indicator and dominating species was *Adansonia* digitata. *Leptadenia hastata, Balanites aegyptiaca, Moringa oleifera, Securidaca longepedunculata, Anogeissus leiocarpa, Saba comorensis, Celosia trigyna,* and *Blyttia fruticulosum* were the dominant associated trees. *Capparis sepiaria, Bridelia scleroneura, Capparis tomentosa, Ziziphus mucronata, Lepidotrichilia volkensii,* and *Sarcocephalus latifolius* were the species that predominate the shrub layer. *Cissus ruspolii, Phytolacca dodecandra, Ampelocissus schimperiana, and Dioscorea bulbifera* were dominant climbers (Table 3).

#### 3.2.3. Community IV (*Anogeissus leiocarpa*-*Periploca linearifolia* Community)

This was positioned at a comparatively greater altitude between 431 and 461 meters above sea level. It was portrayed by 30 plots (1.2 ha) and 105 species (Table 2). The main indicator species in this community was *Anogeissus leiocarpa*. Along with this, *Mimusops kummel, Celosia trigyna,* and *Lonchocarpus laxiflorus* were the prevailing trees in the community. *Capparis sepiaria, Bridelia scleroneura, Asparagus flagellaris,* and *Harrisonia abyssinica* dominate the shrub layer of the community. *Periploca linearifolia, Dorstenia barnimiana,* and *Cissus ruspolii* were the representative climbers of the community (Table 3).

### 3.3. Communities' Species Diversity, Richness, Evenness, and Similarity

Pugnido Forest's overall Shannon–Wiener diversity was 4.41, and the evenness of woody species was 0.93. The four plant communities had a nearly similar diversity index (4.27–4.48), evenness (0.927–0.966), and richness (100–106) (Table 2). Relatively, community IV was the most diverse, whereas community I was the least. The likeness values in species conformation between the communities range from 0.42 to 0.59. The lower resemblance was recorded between communities I and II (0.42%), and the highest resemblance was noted in communities III and IV (0.59%) (Table 4).

### 3.4. Vegetation Structure

#### 3.4.1. Frequency

**Figure 5:**
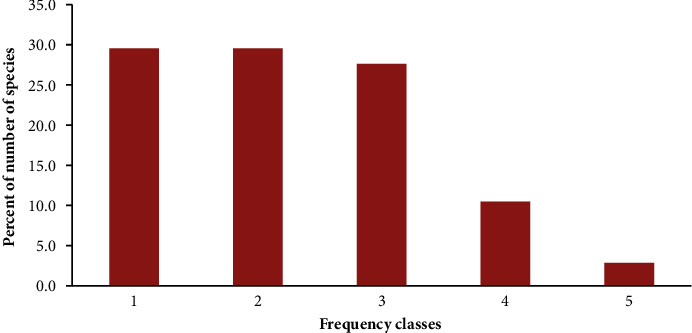
Frequency distribution of woody plant species in Pugnido Forest.

Five frequency classifications, each represented as a percentage, were used to categorize the woody plant species: 1 (≤20), 2 (21–40), 3 (41–60), 4 (61–80), and 5 (81–100). Species were mainly distributed in the first and second classes (29.5% each) that gradually decreased to the higher frequency classes (Figure 5). *Combretum adenogonium* was the most frequently distributed species (88.8%) followed by *Celtis zenkeri* (82.5%) and *Capparis tomentosa* (81.3%). *Acacia bussei* and *Moringa oleifera* were the least frequent species (6.3% each) followed by *Acacia decurrens* and *Acalypha ornata* (7.5% each).

#### 3.4.2. Density

The entire density of the Pugnido forest was 5298.8 stems ha^−1^. Of these, 55.83% were seedlings, whereas 26.78% and 17.39% were saplings and matured plants, respectively. Some species contributed much to the total density of the forest. Among these, *Combretum adenogonium* had the maximum density (240.6 ha^−1^, 4.5%) followed by *Diospyros mespiliformis* (214.1) ha^−1^ (4%), whereas the least dense species recorded were *Moringa oleifera* (2.9 individuals ha^−1^) and *Acalypha ornate* (5.3 individuals ha^−1^) (Table 5).

#### 3.4.3. Diameter at Breast Height (DBH)

**Figure 6:**
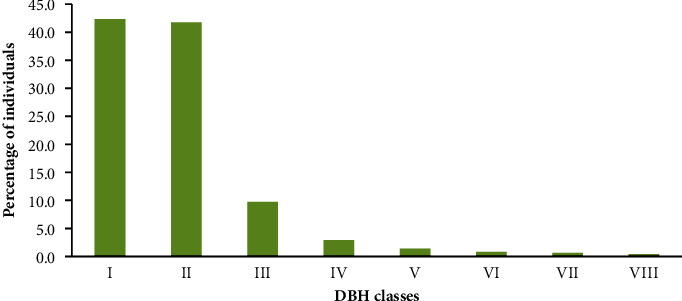
DBH class distribution of species.

In Pugnido Forest, the DBH value of individuals showed decreasing trends from lower to higher classes (Figure 6). The first class contained the majority of individual species (42.3%), followed by the second DBH class (41.8%) and DBH class VIII (0.4%), which had the fewest individuals. *Kigelia aethiopum, Ficus glumosa, Cordia gharaf, Abrus schimperi, Ficus capreifolia,* and *Acacia sieberiana* were the major contributors to the total DBH, whereas *Moringa oleifera, Tapura fischeri,* and *Acalypha ornata* were the least contributors (Table 5).

#### 3.4.4. Tree Height

**Figure 7:**
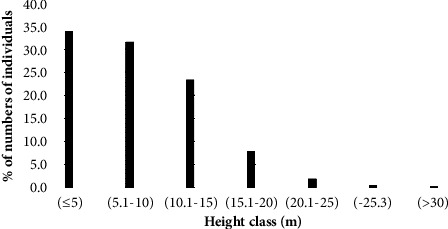
Height class sharing of a species.

The percentage sharing of individuals decreased as height classes increased (Figure 7). Species that contributed to the last height class were *Vitellaria paradoxa* (0.9 ha^−1^, 37.5%), *Terminalia macroptera and Vitex doniana* (0.6 ha^−1^, 25% each), and *Adansonia digitata* (0.3 ha^−1^, 12.5%). *Vitellaria paradoxa* was the emergent (at the top stratum) species with 31 m in height. Higher, middle, and lower story plants were those with heights of >20 m, 10–20 m, and <10 m, respectively, and most (65.8%) species were found in the lower stratum and the least (2.8%) in the top stratum (Table 6).

#### 3.4.5. Basal Area (BA)

**Figure 8:**
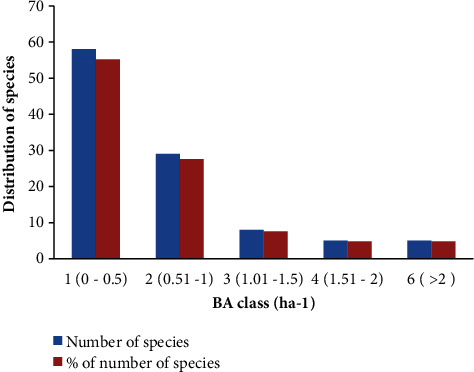
Basal area of mature woody species per hectare.

The entire basal area of all forested species in Pugnido Forest measures 82.5 m^2^·ha^−1^ (Table 5). Most species were dispersed in the foremost class (55.2%), while the last two classes contributed the least (4.8% each) to the total basal area (Figure 8). Species with thigh BA include *Albizia lebbeck* (11.89 ha^−1^, 14.41%), *Kigelia aethiopum* (9.4 ha^−1^, 11.4%), and *Vitellaria paradoxa* (5.27 ha^−1^, 6.39%). In contrast, the lowest basal area (<0.05%) was recorded for species such as *Abrus schimperi, Acalypha ornate,* and *Strychnos innocua* (Table 5).

#### 3.4.6. Importance Value Index (IVI)

From all IVI classes, the uppermost IVI value was observed in species found in class IV (78.7%) followed by class III (11%), while species in class V had the least (0.6%) IVI value (Table 7). Species with a maximum IVI value in the forest include *Albizia lebbeck, Kigelia aethiopum, Combretum adenogonium, Vitellaria paradoxa, Diospyros mespiliformis, Combretum collinum,* and *Combretum molle* (Table 8). A lower IVI value was observed in species of *Leptadenia hastata, Piliostigma thonningii, Acalypha ornate,* and *Moringa oleifera*.

### 3.5. Regeneration Result

The density of seedlings, saplings, and adult woody plant species was found to be 2958.8, 1418, and 921.6 individuals ha^−1^, respectively. The ratio of sapling to seedling, mature to seedling, and mature to sapling woody species was 1 : 2, 1 : 3, and 1 : 2, respectively. At the species level, 100 (94.3%) species had higher seedlings followed by saplings and adult individuals, whereas 4 (3.8%) species including *Vitellaria paradoxa, Tamarindus indica, Kigelia aethiopum,* and *Moringa oleifera* were without seedling stage but had saplings and adult individuals. However, two species (1.9%), *Kigelia aethiopum* and *Moringa oleifera,* had no seedling and sapling stages at all.

## 4. Discussion

### 4.1. Floristic Composition

Pugnido Forest was determined to have higher species richness than Gambella Forest, which has 39 species [14], and lower species richness than Godere Forest, which has 157 species [15], and Gole natural forest, which has 114 species [16]. Such a difference might be due to the physical and edaphic characteristics of the area or different anthropogenic factors. The entire species richness of a given vegetation type indicates the general impression of their diversity [17]. Fabaceae was found to be dominant in several earlier investigations [2, 18–20]. This family's dominance may stem from its excellent ecological adaptation to a variety of climates and its effective systems for pollination and dissemination [21].

### 4.2. Species Diversity, Richness, Evenness, and Similarity of Communities

The existing result indicated that the diversity and consistency of wooded species in Pugnido Forest and the four communities are high as Mekonen et al. [22] described. Kent and Coker [7] also stated that the Shannon–Wiener diversity index is regarded as high if it is beyond 3.0, average if it is 2.0 to 3.0, and low if it is less than 1.0; often fluctuates between 1.5 and 3.5; and seldom surpasses 4.5. All communities show almost identical distribution (richness and consistency). Comparatively, the species diversity and richness of communities III and IV were the highest, but community I had the least. This may be connected with the presence of selective logging in community I as the plots in this community were distributed close to human settlements, which is in agreement with another report [23]. Resemblance coefficients in all community combinations were high (42%–59%). This indicated the existence of relatively high similarity among the recognized community types. This could be observed due to the presence of a nearly similar altitudinal range (431–462 m.a.s.l.), less habitat heterogeneity, and other environmental factors in between the communities.

### 4.3. Frequency, DBH, BA, D, Height, and IVI

Frequency indicates the vegetation homogeneity and vegetation heterogeneity. In Pugnido Forest, lower-frequency classes had a higher species number that showed a decreasing trend towards upper-frequency classes, which is an indicator of excellent species heterogeneity in the forest as Lambrecht [12] described. Parallel results were reported by former authors [24, 25]. The absence of significant external forces and species preferences for their habitats may be the causes of vegetation heterogeneity [25].

Density is a key to wise forest management [26]. Pugnido Forest density (5299 stems ha-^1^) is higher than other forests [26–29]. This could have happened as a result of the forests' varying edaphic, climatic, and manmade causes [28]. Variation in the density of species within the forest might be related to selective external pressure by biotic factors. It might also be a natural characteristic of the species including low seed production, low seed germination, and sensitivity of juveniles to threatening factors, which is an ecological phenomenon.

The DBH class distribution showed a regular inverted J-shaped distribution with a decreasing pattern of species density from lower to higher DBH classes. Other previous similar studies also showed the same pattern [2, 30–32]. The dominance of small-sized individuals in the forest indicated the forest's healthy regeneration and recruitment ability [18].

The total basal area of Pugnido Forest (82.5 m^2^ ha^−1^) was much higher than the average basal area of tropical forests (35 m^2^·ha^−1^) [33]. In addition, the basal area of the study forest was higher than some of the Ethiopian forest patches such as Nech Sar [2], Weiramba [19], Gole [16], and Yemrehane Kirstos Church [20], but it was lesser than Berbera Forest [32], Gelesha Forest [34], Boda Forest [35], and Sese Forest [36]. Many forests in different locations may have varied basal areas due to differences in the forest's age, successional stage, geographic position, or conservation status. The most significant species in the forest are those with the largest basal area [35]. Therefore, the most valuable species in Pugnido Forest was *Albizia lebbeck* followed by *Kigelia aethiopum*, *Vitellaria paradoxa, Ficus capreifolia*, and *Acacia oerfota* in descending order (Table 5).

Using height class analysis, one may comprehend the density of individual plants at various height levels [37] that would lead to judging the successional stage and maturity of the forest. Like DBH class distribution, the height class distributional pattern in Pugnido Forest experienced an inverted J-shaped pattern in which the lower height class had a greater distribution of individuals and vice versa. Previous research revealed similar findings in Ethiopia [14, 31, 38]. This suggested that to comprehend the density of individual plants in relation to size, DBH and height are crucial components as Masresha and Melkamu [37] described. The occurrence of *Vitellaria paradoxa* at the top canopy followed by *Terminalia macroptera, Vitex doniana,* and *Adansonia digitata* could be related to their adaptation potential to climatic and edaphic factors. It might also be the presence of special conservation precedence for the species by the local people related to their other ethnobotanical values.

Importance value index (IVI) is useful to differentiate each species' ecological relevance [12] in which species' social structure in the community is high when the IVI value is high. Therefore, the leading species such as *Albizia lebbeck, Kigelia aethiopum,* and *Combretum adenogonium* might be the most adaptable and successful species that resist external and internal disturbances with good reproduction, regeneration, and recruitment as described by Belachew [36], but lower IVI values of some species might be related to their poor adaptability for the natural ecology of the area.

### 4.4. Plant Species Regeneration Status

If forest seedlings are greater than saplings and then mature plants, their regeneration level is termed healthy [32]. Therefore, the current result confirmed that Pugnido Forest is at a good regeneration status. Similar results were reported by different previous forest studies in Ethiopia [2, 16, 20]. At the species level, most (94.7%) species showed good regeneration status, whereas some (3.5%) species showed poor regeneration. However, 2 (1.9%) species (*Kigelia aethiopum and Moringa oleifera*) were not regenerating. According to Shibru and Balcha [8], protection precedence ought to be provided for species with no or only one seedling followed by seedlings greater than one but lower than fifteen. Accordingly, immediate conservation concern should be given for six species (*Kigelia aethiopum, Moringa oleifera, Vitellaria paradoxa, Tamarindus indica, Kigelia aethiopum,* and *Moringa oleifera*) before their total loss.

## 5. Conclusions

The high overall plant diversity, evenness, and richness of the study forest demonstrated the relative stability and healthier ecological condition of the forest ecosystem serving as an *in situ* conservation site. According to structural data analyses (DBH, height, and BA classes), the forest is largely conquered by small trees and shrubs, which suggests the existence of previous disturbance or secondary nature of the forest. Some species have no or few numbers of seedlings indicating their risky level of local extinction unless urgent remedial actions are taken. Therefore, conserving the species in seed banks or planting the species through nursery production is needed.

## Figures and Tables

**Table 1:** List of woody plant species collected from Pugnido Forest.

Species	Families	Habit
*Abrus precatorius* L.	Fabaceae	Climber
*Abrus schimperi* Hochst. ex Bak.	Fabaceae	Shrub
*Abutilon mauritianum* (Jacq.) Medik	Malvaceae	Shrub
*Acacia albida* Del.	Fabaceae	Tree
*Acacia brevispica* Harms	Fabaceae	Tree
*Acacia bussei* Harms ex Sjöstedt	Fabaceae	Tree
*Acacia decurrens* Wild	Fabaceae	Tree
*Acacia lahai* Steud & Hochst. ex Benth	Fabaceae	Tree
*Acacia melanoxylon* R. Br.	Fabaceae	Tree
*Acacia mearnsii* De Wild	Fabaceae	Tree
*Acacia nilotica* (L.) Wild. ex Del.	Fabaceae	Tree
*Acacia oerfota* (Forssk.) Schweinf.	Fabaceae	Shrub
*Acacia polyacantha* Willd.	Fabaceae	Tree
*Acacia Senegal* (L.) Wild	Fabaceae	Tree
*Acacia seyal* Del.	Fabaceae	Tree
*Acacia sieberiana* DC.	Fabaceae	Tree
*Acalypha acrogyna* Pax	Euphorbiaceae	Shrub
*Acalypha ornata* A. Rich	Euphorbiaceae	Shrub
*Acokanthera schimperi* (A. DC.) Schweinf.	Apocynaceae	Shrub
*Adansonia digitata* L.	Malvaceae	Tree
*Albizia grandibracteata* Taub.	Fabaceae	Tree
*Albizia lebbeck* (L.) Benth	Fabaceae	Tree
*Albizia malacophylla* (A. Rich.) Walp.	Fabaceae	Tree
*Albizia gummifera* (JF. Gmel.) CA. Sm.	Fabaceae	Tree
*Alstonia boonei* De Wild	Malvaceae	Tree
*Ampelocissus schimperiana* (Hochst. ex A. Rich) Planch.	Vitaceae	Climber
*Annona senegalensis* Pers.	Annonaceae	Tree
*Anogeissus leiocarpa* (A. DC.) Guill. & Perr.	Combretaceae	Tree
*Antiaris toxicaria* lesch	Moraceae	Tree
Argomuellera macrophylla Pax	Euphorbiaceae	Tree
*Asparagus flagellaris* (Kunth) Baker	Asparagaceae	Shrub
*Balanites aegyptiaca* (L.) Del.	Balanitaceae	Tree
*Baphia abyssinica* Brummitt	Fabaceae	Tree
*Blythia fruticosum* (Decne.) D.V.Field	Asclepiadaceae	Tree
*Borassus aethiopum* Mart.	Arecaceae	Tree
*Bridelia scleroneura* Muell. Arg.	Euphorbiaceae	Shrub
*Capparis erythrocarpos* Isert	Capparidaceae	Shrub
*Capparis sepiaria* L.	Capparidaceae	Shrub
*Capparis tomentosa* Lam	Capparidaceae	Shrub
*Celtis toka* (Forssk.) Hepper & Wood	Ulmaceae	Tree
*Celtis zenkeri* Engl.	Ulmaceae	Tree
*Cissampelos mucronata* A. Rich.	Menispermaceae	Climber
*Cissus ruspolii* Gilg	Vitaceae	Climber
*Combretum adenogonium* Steud. ex A. Rich.	Combretaceae	Tree
*Combretum collinum* Fresen	Combretaceae	Tree
*Combretum molle* R. Br. ex G. Don	Combretaceae	Tree
*Cordia africana* Lam	Boraginaceae	Tree
*Cordia gharaf* (Forssk.) Aschers.	Boraginaceae	Tree
*Dioscorea praehensilis* Benth	Dioscoreaceae	Climber
*Diospyros abyssinica* (Hiern) F. White	Ebenaceae	Tree
*Diospyros mespiliformis* Hochst. ex A.DC.	Ebenaceae	Tree
*Entada africana* Guill. & Perr.	Fabaceae	Tree
*Erythroxylum fischeri Engl.*	Erythroxylaceae	Tree
*Ficus capreifolia* Del.	Moraceae	Tree
*Ficus dicranostyla* Mildbr.	Moraceae	Tree
*Ficus glumosa Del.*	Moraceae	Tree
*Ficus sycomorus* L.	Moraceae	Tree
*Ficus thonningii* Blume	Moraceae	Tree
*Ficus vasta* Forssk.	Moraceae	Tree
*Flueggea virosa* (Willd.) Voigt.	Phyllanthaceae	Shrub
*Gardenia ternifolia* Schumach. & Thonn.	Rubiaceae	Tree
*Grewia mollis* A. Juss.	Tiliaceae	Tree
*Grewia velutina* A. Rich.	Tiliaceae	Tree
*Harrisonia abyssinica* Oliv.	Simaroubaceae	Shrub
*Loeseneriella africana* (Willd.) Loes.	Celastraceae	Climber
*Jatropha curcas* L.	Euphorbiaceae	Tree
*Kigelia aethiopum* L.	Bignoniaceae	Tree
*Lannea fruticosa* (A.Rich.) Engl.	Anacardiaceae	Tree
*Lepidotrichilia volkensii* (Gürke) Leroy	Meliaceae	Shrub
*Leptadenia hastata* (Pers.) Decne.	Asclepiadaceae	Climber
*Lonchocarpus laxiflorus* Guill & Perr.	Fabaceae	Tree
*Malacantha alnifolia* (Bak.) Pierre	Sapotaceae	Tree
*Milicia excelsa* (Welw.) C.C. Berg	Moraceae	Tree
*Mimusops kummel* A. De.	Sapotaceae	Tree
*Moringa oleifera* Lam	Moringaceae	Tree
*Morus mesozygia* Stapf	Moraceae	Tree
*Oncoba spinosa* Forssk.	Flacourtiaceae	Tree
*Oxyanthus lepidus* S. Moore	Rubiaceae	Shrub
*Periploca linearifolia* Quart.-Dill. & A. Rich.	Asclepiadaceae	Climber
*Phytolacca dodecandra* L′Herit	Phytolaccaceae	Climber
*Piliostigma thonningii* (Schumach.) Milne-Redh	Fabaceae	Shrub
*Pouteria alnifolia* (Bak.) Roberty	Sapotaceae	Tree
*Pseudocedrela kotschyi* (Schweinf.) Harms	Meliaceae	Tree
*Pterocarpus lucens* Guill. & Perr.	Fabaceae	Tree
*Saba comorensis* (Bol.) Pichon	Apocynaceae	Tree
*Sarcocephalus latifolius* (Smith) Bruce	Rubiaceae	Shrub
*Sclerocarya birrea* (A. Rich.) Hochst.	Anacardiaceae	Tree
*Securidaca longepedunculata* Fresen	Polygalaceae	Tree
*Sterculia africana* (Lour.) Fiori	Sterculiaceae	Tree
*Stereospermum kunthianum* Cham	Bignoniaceae	Tree
*Strychnos innocua* Del.	Loganiaceae	Tree
*Syzygium guineense* (Willd.) De.	Myrtaceae	Tree
*Tamarindus indica* L.	Fabaceae	Tree
*Tapinanthus heteromorphus* (A. Rich.) Danser	Loranthaceae	Shrub
*Tapura fischeri* Engl.	Dichapetalaceae	Tree
*Terminalia laxiflora* Engl. & Diels	Combretaceae	Tree
*Terminalia macroptera* Guill. & Perr.	Combretaceae	Tree
*Turraea nilotica* Kotschy & Peyr.	Meliaceae	Tree
*Vangueria apiculata* K. Schum.	Rubiaceae	Tree
*Vepris dainellii* (Pic.Serm.) Kokwaro	Rutaceae	Tree
*Vitellaria paradoxa* Gaertn.f.	Sapotaceae	Tree
*Vitex doniana* Sweet	Verbenaceae	Tree
*Xylopia parviflora* (A. Rich.) Benth	Annonaceae	Tree
*Ziziphus abyssinica* Hochst. ex A. Rich.	Rhamnaceae	Shrub
*Ziziphus mucronata* Willd.	Rhamnaceae	Shrub
*Ziziphus pubescens* Oliv.	Rhamnaceae	Tree

**Table 2:** Plant communities' species evenness, richness, and diversity index.

Community	Species richness	Diversity index (H)	H′ max	Species evenness
I	98	4.27	4.61	0.927
II	104	4.42	4.70	0.955
II	106	4.46	4.74	0.956
IV	106	4.48	4.74	0.966

**Table 3:** Species having synoptic value greater than 1 in one or more communities.

Species	Cluster 1	Cluster 2	Cluster 3	Cluster 4
*Acacia albida*	2.6	0.23	1.11	0.9
*Acacia brevispica*	0.2	2.38	1.33	1.1
*Acacia bussei*	0.6	1.38	0.41	1.33
*Acacia decurrens*	2.2	0.15	0.59	0.17
*Acacia lahai*	1.1	0.46	0.56	0.57
*Acacia melanoxylon*	3.3	2.23	0.89	0.2
*Acacia mearnsii*	1.4	1.08	0.3	1.03
*Acacia nilotica*	0	0.31	1.33	1.27
*Acacia senegal*	1	1.54	1.67	1
*Acacia sieberiana*	0.6	0.62	0.56	1.37
*Acokanthera schimperi*	0.5	1.08	1.56	0.23
*Adansonia digitata*	0.2	0	3.48	0.53
*Albizia grandibracteata*	1	0.85	1.15	0.73
*Albizia lebbeck*	0.7	4.69	0.81	1.73
*Albizia malacophylla*	0	0.54	0.48	1.77
*Albizia gummifera*	2.3	2.38	1.33	0.8
*Alstonia boonei*	1.7	0.92	0.15	0.73
*Ampelocissus schimperiana*	0	0.31	1.33	0.5
*Annona senegalensis*	0.1	0.62	0.26	1.47
*Anogeissus leiocarpa*	1.2	0.69	1.78	5.57
*Antiaris toxicaria*	1.3	2.15	1.41	1.27
*Argomuellera macrophylla*	2.9	0.62	1.52	1.13
*Balanites aegyptiaca*	5	1.23	2.3	1.9
*Baphia abyssinica*	1.8	2.15	0.33	0.37
*Blyttia fruticulosum*	0.3	0.85	1.7	1.4
*Borassus aethiopum*	1.8	1.08	0.56	1.07
*Bridelia scleroneura*	1.7	1	3.04	2.2
*Capparis erythrocarpos*	0.7	0.15	0.78	1.27
*Capparis sepiaria*	7.4	1.46	2.74	2.7
*Capparis tomentosa*	0.7	1.77	2.15	1.07
*Celosia trigyna*	1.3	0.85	1.74	2.67
*Celtis toka*	0.9	1.54	0.63	1.33
*Celtis zenkeri*	0.9	1.31	1.15	1.83
*Cissus ruspolii*	2.3	0.85	1.93	1.87
*Combretum adenogonium*	0.8	2.31	1.19	1.33
*Combretum collinum*	0.4	0	0.81	1.77
*Combretum molle*	0.9	0.92	0.89	1.63
*Cordia africana*	0.8	0.62	1.63	1.57
*Dioscorea bulbifera*	0.8	3.08	1.04	0.8
*Dioscorea praehensilis*	0.3	0.69	0.26	1.33
*Diospyros abyssinica*	1.9	0.77	0.89	0.53
*Diospyros mespiliformis*	1.5	0.54	0.74	1.2
*Dorstenia barnimiana*	0.3	1.23	0.44	1.9
*Entada africana*	0.5	1.62	0.11	0.6
*Erythroxylum fischeri*	0	0.54	0.74	1.4
*Ficus capreifolia*	2	0.23	1.37	1.17
*Ficus dicranostyla*	0	0.54	0.7	1.77
*Ficus glumosa*	1.3	0.62	0.52	0.9
*Ficus sycomorus*	1.7	0.62	0.93	0.8
*Ficus thonningii*	0.5	1.08	1.37	1
*Ficus vasta*	0.4	1	0.89	1.3
*Flueggea virosa*	0.4	1	0.52	1.57
*Gardenia ternifolia*	0	1.31	0.63	0.9
*Grewia mollis*	0.4	0.54	0.11	1.47
*Grewia velutina*	1.9	1.08	0.63	1.07
*Harrisonia abyssinica*	0.1	0.69	0.19	2.07
*Hippocratea africana*	0.5	0.62	0.85	1.3
*Kigelia aethiopum*	0	0.92	1.59	1.03
*Lepidotrichilia volkensii*	0.5	0.54	1.37	0.7
*Leptadenia hastate*	0.5	0.15	2.44	1.5
*Lonchocarpus laxiflorus*	0.3	0.31	0.26	2.23
*Malacantha alnifolia*	2	0.69	1.44	1.1
*Mimusops kummel*	0.2	1.46	0.93	2.7
*Moringa oleifera*	0.1	2	2.11	1.2
*Morus mesozygia*	0.6	0.62	1.33	1.2
*Oxyanthus lepidus*	1	1.23	0.56	1.97
*Periploca linearifolia*	0.5	0.92	0.52	2.87
*Phytolacca dodecandra*	0.9	0.62	1.22	0.83
*Piliostigma thonningii*	0.3	1.85	0.78	1.5
*Pouteria alnifolia*	1	0.54	0.3	0.83
*Pseudocedrela kotschyi*	0	2.15	0.85	0.43
*Pterocarpus lucens*	0.9	1	0.96	1.2
*Saba comorensis*	1.6	0.69	1.78	1.2
*Sarcocephalus latifolius*	1.1	1.69	0.96	1.63
*Sclerocarya birrea*	0.8	1.46	0.52	1.13
*Securidaca longepedunculata*	0.9	0.92	1.93	0.9
*Sterculia africana*	0.6	1.15	0.74	0.7
*Stereospermum kunthianum*	1.3	1.46	1.41	0.7
*Syzygium guineense*	0.8	1.46	1.22	0.77
*Tamarindus indica*	0.3	2.23	0.89	1.33
*Tapinanthus heteromorphus*	1.3	0.85	0.7	0.87
*Terminalia laxiflora*	2	0.38	0.7	0.47
*Vepris dainellii*	1.8	0.69	0.67	1.03
*Vitellaria paradoxa*	0.7	2	0.48	1.13
*Vitex doniana*	1.9	0.85	1.04	0.57
*Ziziphus abyssinica*	1.7	0.85	0.63	0.5
*Ziziphus mucronata*	0.2	1.69	2.11	0.47

**Table 4:** Similarity between the communities in species composition.

Communities	I	II	III	IV
I				
II	0.42			
III	0.43	0.57		
IV	0.44	0.57	0.59	

**Table 5:** Density (D), DBH (in m), and basal area (BA, in m^2^·ha^−1^) of all woody plant species.

Species	D	DBH	BA
*Abrus schimperi*	90.6	20	0.00
*Abutilon mauritianum*	60.3	9.3	0.28
*Acacia Senegal*	37.5	16.6	0.73
*Acacia albida*	53.8	15.3	0.39
*Acacia decurrens*	56.6	7.2	0.67
*Acacia lahai*	58.8	4.1	0.19
*Acacia melanoxylon*	15.0	9.8	1.09
*Acacia nilotica*	30.6	6	0.39
*Acacia polyacantha*	64.1	14.7	1.69
*Acacia bussei*	69.4	6.3	0.00
*Acacia seyal*	28.1	7.2	0.62
*Acacia brevispica*	115.	6.6	0.50
*Acacia oerfota*	35.3	16.7	2.04
*Acacia sieberiana*	45.6	18.1	1.34
*Acacia mearnsii*	54.4	9.7	0.57
*Acalypha acrogyna*	16.9	7.2	0.36
*Acalypha ornata*	5.3	0.9	0.00
*Acokanthera schimperi*	9.4	6.3	0.74
*Adansonia digitata*	43.8	12.8	0.71
*Albizia grandibracteata*	15.9	6	0.51
*Albizia lebbeck*	15.9	9.8	11.9
*Albizia malacophylla*	20.3	5.9	0.30
*Albizia gummifera*	33.8	5.9	0.40
*Alstonia boonei*	28.8	9.3	0.59
*Amaranthus spinosus*	12.8	7.5	0.35
*Ampelocissus schimperiana*	2.16	0.00	0.00
*Annona senegalensis*	125	5.9	0.58
*Anogeissus leiocarpa*	103	5.9	0.21
*Antiaris toxicaria*	31.3	9.7	0.44
*Argomuellera macrophylla*	61.3	6.9	0.28
*Asparagus flagellaris*	1294	12.6	0.70
*Balanites aegyptiaca*	58.8	8.5	0.42
*Baphia abyssinica*	59.4	5.9	0.40
*Blyttia fruticulosum*	67.2	5.9	0.27
*Borassus aethiopum*	100	6.3	0.54
*Bridelia scleroneura*	36.3	8.8	0.77
*Bulbostylis clarkeana*	21.6	7.5	0.33
*Capparis sepiaria*	41.6	6.9	0.63
*Capparis erythrocarpos*	20.9	10.6	0.47
*Capparis tomentosa*	57.5	12.5	0.42
*Celosia trigyna*	40.0	11	0.60
*Celtis toka*	75.9	15.9	0.72
*Celtis zenkeri*	90.3	13.8	0.71
*Cissampelos mucronata*	148	17.6	0.79
*Combretum adenogonium*	241	15	1.11
*Combretum collinum*	157	9.7	0.33
*Combretum molle*	123	12.2	0.76
*Cordia africana*	19.7	12	1.15
*Cordia gharaf*	44.7	20.7	1.64
*Diospyros abyssinica*	62.8	6.9	0.22
*Diospyros mespiliformis*	214	14.4	0.98
*Entada africana*	23.4	5.7	0.62
*Erythroxylum fischeri*	14.7	6.5	0.45
*Ficus capreifolia*	24.1	18.2	2.81
*Ficus glumosa*	27.5	20.5	1.49
*Ficus sycomorus*	25.6	14.6	1.98
*Ficus thonningii*	55.9	9	0.65
*Ficus vasta*	15.0	15.5	1.02
*Flueggea virosa*	40.9	10.9	0.72
*Gardenia ternifolia*	53.1	17.4	0.82
*Grewia mollis*	51.3	6.3	0.17
*Grewia velutina*	58.1	6.5	0.27
*Harrisonia abyssinica*	30.6	6.2	0.22
*Indigofera arrecta*	24.1	8	0.25
*Jatropha curcas*	19.4	5.9	0.07
*Kigelia aethiopum*	14.1	22.3	9.40
*Lannea fruticosa*	24.4	10	0.33
*Lepidotrichilia volkensii*	57.8	6.6	0.18
*Leptadenia hastata*	20.3	8.7	0.03
*Lonchocarpus laxiflorus*	94.1	6.9	0.31
*Malacantha alnifolia*	68.4	6	0.18
*Milicia excelsa*	23.4	9.3	0.65
*Mimusops kummel*	25.0	18.3	0.21
*Momordica foetida*	23.4	14.7	1.87
*Moringa oleifera*	0.9	0.9	0.01
*Morus mesozygia*	47.5	5.9	0.57
*Oncoba spinosa*	15.3	5.6	1.51
*Oxyanthus lepidus*	78.4	5.6	1.32
*Periploca linearifolia*	97.5	5.6	0.27
*Ficus dicranostyla*	22.2	2.4	0.20
*Piliostigma thonningii*	8.8	5.7	0.09
*Pouteria alnifolia*	16.9	6.3	0.93
*Pseudocedrela kotschyi*	22.8	6.3	0.50
*Pterocarpus lucens*	60.3	5.6	0.19
*Saba comorensis*	120.0	5.6	0.22
*Sarcocephalus latifolius*	12.5	5.3	0.18
*Sclerocarya birrea*	35.9	5.6	0.57
*Securidaca longepedunculata*	63.1	5.3	0.06
*Sterculia africana*	58.8	5.7	0.73
*Stereospermum kunthianum*	61.9	5.6	0.15
*Strychnos innocua*	57.8	5.6	0.00
*Syzygium guineense*	25.6	5.3	0.08
*Tamarindus indica*	6.6	5.3	0.06
*Tapinanthus heteromorphus*	30.3	5.3	0.04
*Tapura fischeri*	24.4	1.9	0.01
*Terminalia laxiflora*	81.9	5.6	0.00
*Terminalia macroptera*	94.4	5.6	0.74
*Terraria nilotica*	49.4	5.3	0.02
*Vangueria apiculata*	16.6	5.3	0.31
*Vepris dainellii*	49.1	5	0.03
*Vitellaria paradoxa*	12.8	10.9	5.27
*Vitex doniana*	6.6	5.7	0.47
*Xylopia parviflora*	93.4	5.6	0.08
*Ziziphus abyssinica*	25.3	5.6	0.08
*Ziziphus mucronata*	21.3	8.2	1.07
*Ziziphus pubescens*	18.4	5.9	0.25
Total	5299		82.5

**Table 6:** Vertical stratification of the forest species.

Story	Stem number (ha^−1^)	% Stem number (ha^−1^)	Species number	% Species number	Individuals to species ratio (ha^−1^)
Lower	594.1	65.8	101	48.8	5.9 : 1
Middle	284.4	31.5	91	44.0	3.1 : 1
Upper	25	2.8	15	7.2	1.7 : 1

**Table 7:** IVI class and species number in each class.

IVI class	Species count	IVI total	IVI percentage
V	4	1.9	0.6
IV	94	235.3	78.7
III	5	32.9	11.0
II	1	13.69	4.6
I	2	15.42	5.2

**Table 8:** Woody species IVI and priority class for conservation in Pugnido Forest.

Species	*F* (%)	RD	RDO	RFR	IVI	Rank	Priority class
*Albizia lebbeck*	25	0.30	14.41	0.71	15.42	1	1
*Kigelia aethiopum*	75	0.27	11.40	2.02	13.69	2	2
*Combretum adenogonium*	88.8	4.54	1.34	2.39	8.27	3	3
*Vitellaria paradoxa*	23.8	0.24	6.39	0.64	7.27	4	3
*Diospyros mespiliformis*	55	4.04	1.19	1.48	6.71	5	3
*Combretum collinum*	80	2.95	0.40	2.15	5.50	6	3
*Combretum molle*	70	2.32	0.92	1.88	5.12	7	3
*Ficus capreifolia*	27.5	0.45	3.41	0.74	4.60	8	4
*Celtis zenkeri*	82.5	1.70	0.87	1.85	4.42	9	4
*Asparagus flagellaris*	41.3	2.44	0.85	1.11	4.40	10	4
*Cordia gharaf*	56.3	0.84	1.99	1.51	4.34	11	4
*Cissampelos mucronata*	16.3	2.78	0.96	0.44	4.18	12	4
*Ficus glumosa*	68.8	0.52	1.81	1.85	4.18	13	4
*Celtis toka*	68.8	1.43	0.87	1.85	4.15	14	4
*Terminalia macroptera*	31.3	1.78	0.90	1.28	3.96	15	4
*Oxyanthus lepidus*	28.5	1.48	1.60	0.77	3.85	16	4
*Saba comorensis*	47.5	2.26	0.26	1.28	3.80	17	4
*Capparis tomentosa*	81.3	1.09	0.51	2.18	3.78	18	4
*Anogeissus leiocarpa*	56.3	1.96	0.26	1.51	3.73	19	4
*Acacia polyacantha*	16.3	1.21	2.05	0.44	3.70	20	4
*Lonchocarpus laxiflorus*	56.3	1.78	0.38	1.51	3.67	21	4
*Ficus sycomorus*	31.3	0.48	2.24	0.84	3.56	22	4
*Stereospermum kunthianum*	78.5	1.17	0.18	2.12	3.47	23	4
*Acacia oerfota*	18.8	0.67	2.24	0.50	3.41	24	4
*Capparis sepiaria*	68.8	0.78	0.77	1.85	3.40	25	4
*Annona senegalensis*	10	2.36	0.70	0.27	3.33	26	4
*Acacia brevispica*	13.8	2.17	0.61	0.37	3.15	27	4
*Borassus aethiopum*	22.5	1.89	0.65	0.60	3.14	28	4
*Balanites aegyptiaca*	56.3	1.11	0.51	1.51	3.13	29	4
*Oncoba spinosa*	35	0.29	1.83	0.94	3.06	30	4
*Entada africana*	68.8	0.44	0.75	1.85	3.04	31	4
*Sterculia africana*	36.3	1.11	0.89	0.97	2.97	32	4
*Ficus thonningii*	41.3	1.06	0.79	1.11	2.96	33	4
*Cordia africana*	43.8	0.37	1.39	1.18	2.94	34	4
*Acacia sieberiana*	15	0.86	1.62	0.40	2.88	35	4
*Diospyros abyssinica*	51.3	1.19	0.27	1.38	2.84	37	4
*Terminalia laxiflora*	47.5	1.55	0.01	1.28	2.84	38	4
*Lepidotrichilia volkensii*	56.3	1.09	0.21	1.51	2.81	39	4
*Blyttia fruticulosum*	42.5	1.27	0.33	1.14	2.74	40	4
*Bridelia scleroneura*	41.3	0.68	0.93	1.11	2.72	41	4
*Lannea fruticosa*	68.8	0.46	0.40	1.85	2.71	42	4
*Ficus vasta*	42.5	0.28	1.23	1.14	2.65	43	4
*Malacantha alnifolia*	42.5	1.29	0.22	1.14	2.65	44	4
*Sclerocarya birrea*	47.5	0.68	0.69	1.28	2.65	45	4
*Xylopia parviflora*	28.5	1.76	0.10	0.77	2.63	46	4
*Celosia trigyna*	42.5	0.75	0.72	1.14	2.61	47	4
*Gardenia ternifolia*	22.5	1.00	0.99	0.60	2.59	48	4
*Periploca linearifolia*	13.8	1.84	0.33	0.37	2.54	49	4
*Flueggea virosa*	32.5	0.77	0.87	0.87	2.51	50	4
*Tapinanthus heteromorphus*	60	0.57	0.31	1.61	2.49	51	4
*Antiaris toxicaria*	48.8	0.59	0.53	1.31	2.43	52	4
*Bulbostylis clarkeana*	57.5	0.41	0.39	1.55	2.35	53	4
*Ziziphus mucronata*	23.8	0.40	1.30	0.64	2.34	54	4
*Strychnos innocua*	46.3	1.09	0.00	1.24	2.33	55	4
*Adansonia digitata*	23.8	0.83	0.86	0.64	2.33	56	4
*Tamarindus indica*	73.8	0.12	0.07	2.12	2.31	57	4
*Ficus dicranostyla*	61.3	0.42	0.24	1.65	2.31	58	4
*Acacia Senegal*	23.8	0.71	0.88	0.64	2.23	59	4
*Acacia melanoxylon*	21.3	0.28	1.32	0.57	2.17	60	4
*Acacia mearnsii*	16.3	1.03	0.69	0.44	2.16	61	4
*Pseudocedrela kotschyi*	41.3	0.43	0.60	1.11	2.14	62	4
*Securidaca longepedunculata*	32.5	1.19	0.07	0.87	2.13	63	4
*Alstonia boonei*	31.3	0.54	0.72	0.84	2.10	64	4
*Argomuellera macrophylla*	22.5	1.16	0.34	0.60	2.10	65	4
*Acacia albida*	22.5	1.01	0.48	0.60	2.09	66	4
*Syzygium guineense*	56.3	0.48	0.09	1.51	2.08	67	4
*Acacia decurrens*	7.5	1.07	0.81	0.20	2.08	68	4
*Morus mesozygia*	17.5	0.90	0.69	0.47	2.06	69	4
*Abrus schimperi*	12.5	1.71	0.00	0.34	2.05	70	4
*Albizia gummifera*	33.8	0.64	0.48	0.91	2.03	71	4
*Baphia abyssinica*	15	1.12	0.48	0.40	2.00	72	4
*Abutilon mauritianum*	18.8	1.14	0.34	0.50	1.98	73	4
*Milicia excelsa*	27.5	0.44	0.78	0.74	1.96	74	4
*Pouteria alnifolia*	18.8	0.32	1.13	0.50	1.95	75	4
*Tapura fischeri*	53.8	0.46	0.01	1.44	1.91	76	4
*Vitex doniana*	43.8	0.12	0.57	1.18	1.87	77	4
*Pterocarpus lucens*	18.8	1.14	0.23	0.50	1.87	78	4
*Grewia mollis*	25	0.97	0.21	0.67	1.85	79	4
*Acacia lahai*	18.8	1.11	0.23	0.50	1.84	80	4
*Vangueria apiculata*	42.5	0.31	0.37	1.14	1.82	81	4
*Amaranthus spinosus*	41.3	0.24	0.42	1.11	1.77	82	4
*Grewia velutina*	12.5	1.10	0.33	0.34	1.77	83	4
*Albizia malacophylla*	32.5	0.38	0.36	0.87	1.61	84	4
*Acacia seyal*	11.3	0.53	0.75	0.30	1.58	85	4
*Capparis erythrocarpos*	22.5	0.40	0.57	0.60	1.57	86	4
*Indigofera arrecta*	28.5	0.45	0.31	0.77	1.53	87	4
*Acacia nilotica*	17.5	0.58	0.47	0.47	1.52	88	4
*Ziziphus pubescens*	42.5	0.35	0.03	1.14	1.52	89	4
*Acacia bussei*	6.3	1.31	0.01	0.17	1.49	90	4
*Vepris dainellii*	18.8	0.93	0.04	0.50	1.47	91	4
*Terraria nilotica*	18.8	0.93	0.03	0.50	1.46	92	4
*Albizia grandibracteata*	17.5	0.30	0.61	0.47	1.38	93	4
*Harrisonia abyssinica*	18.8	0.58	0.27	0.50	1.35	94	4
*Acokanthera schimperi*	10	0.18	0.90	0.27	1.35	95	4
*Mimusops kummel*	21.3	0.47	0.26	0.57	1.30	96	4
*Erythroxylum fischeri*	17.5	0.28	0.55	0.47	1.30	97	4
*Sarcocephalus latifolius*	28.5	0.24	0.22	0.77	1.23	98	4
*Ziziphus abyssinica*	21.3	0.48	0.10	0.57	1.15	99	4
*Jatropha curcas*	21.3	0.37	0.08	0.57	1.02	100	4
*Acalypha acrogyna*	8.8	0.32	0.44	0.24	1.00	101	4
*Leptadenia hastata*	12.5	0.38	0.04	0.34	0.76	102	5
*Piliostigma thonningii*	12.5	0.17	0.07	0.34	0.58	103	5
*Acalypha ornata*	7.5	0.10	0.00	0.20	0.30	104	5
*Moringa oleifera*	6.3	0.02	0.02	0.17	0.21	105	5

## Data Availability

The entire data collected for this study were analyzed, interpreted, and incorporated into this article.
